# A Behavioral and Electrophysiological Investigation of the Effect of Bilingualism on Lexical Ambiguity Resolution in Young Adults

**DOI:** 10.3389/fnhum.2015.00682

**Published:** 2015-12-21

**Authors:** Shanna Kousaie, Christianne Laliberté, Rocío López Zunini, Vanessa Taler

**Affiliations:** ^1^Bruyère Research InstituteOttawa, ON, Canada; ^2^School of Psychology, Faculty of Social Sciences, University of OttawaOttawa, ON, Canada

**Keywords:** lexical ambiguity processing, homonyms, event-related brain potentials (ERPs), N400, bilingualism

## Abstract

Previous research suggests that bilinguals demonstrate superior cognitive control processes than monolinguals. The goal of the current investigation was to examine whether this “bilingual advantage” is observed in a language processing task that requires inhibition, i.e., lexical ambiguity processing. Monolingual and bilingual participants read sentences that biased the reading of a terminal homonym toward the subordinate or dominant reading (e.g., The doctor asked her to step onto the scale.). A relatedness judgment was made on target words that were related to the contextually appropriate (e.g., balance) or inappropriate meaning (e.g., skin), or unrelated to either meaning (e.g., shoe) while electrophysiological recording took place. The results revealed subtle processing differences between monolinguals and bilinguals that were evident in electrophysiological measures, but not in behavioral measures. These findings suggest that monolinguals rely on context to access the contextually appropriate meaning of a homonym to a greater extent than bilinguals, while bilinguals demonstrate simultaneous activation of both meanings.

## Introduction

Language is our primary form of communication and involves many cognitive processes. Furthermore, more than 50% of the world's population is bilingual or multilingual, meaning that these individuals manage more than one language (e.g., Grosjean, 1989, 2008; Fabbro, 1999). Bilingualism and the cognitive consequences of being bilingual are areas of research that have received an increased amount of interest over the last decade. Some findings suggest that bilingual individuals demonstrate superior cognitive function relative to their monolingual peers (e.g., Bialystok et al., 2005; Bialystok, 2006; Costa et al., 2008), despite disadvantages in vocabulary size in children and on measures of lexical access/retrieval in adults (see Bialystok, 2009). Findings demonstrating superior performance for bilinguals endorse what has become known as the “bilingual advantage” hypothesis, which postulates that the constant management of two (or more) languages results in superior and more robust cognitive control processes, including inhibition, switching, and working memory (Bialystok et al., 2012).

The bilingual advantage refers to findings showing that on tasks requiring attentional/cognitive control, such as the Stroop (Stroop, 1935), Simon (Simon and Rudell, 1967), and Flanker (Eriksen and Eriksen, 1974) tasks, bilinguals demonstrate superior performance relative to monolinguals (i.e., faster response times overall and/or smaller interference effects). The common element in these tasks is the presence of both target-relevant and target-irrelevant information that can be incongruent in terms of the response that they prompt. The bilingual advantage hypothesis purports that the constant management of two languages by bilinguals requires general cognitive control mechanisms to select the appropriate language, suppress intrusions from the non-target language, and to effortlessly switch between languages, which is thought to result in more efficient and robust cognitive control processes (see Bialystok et al., 2012). However, support for this hypothesis is not universal. For example, Kousaie and Phillips (2012a) have failed to find any differences between monolingual and bilingual young and older adults using a behavioral Stroop task. In a second study, Kousaie and Phillips (2012b) found language group difference on electrophysiological measures of cognitive control, but not on behavioral measures, making it impossible to conclude that there was a bilingual advantage. That is, given that there were no differences in behavior indicating superior performance by the bilinguals, it is unclear whether the observed electrophysiological differences represent an advantage or simply a different neural response leading to the same outcome. Others have also failed to find consistent differences between monolinguals and bilinguals (for a review and alternate view of the bilingual advantage see Hilchey and Klein, 2011; Paap and Greenberg, 2013; Paap and Sawi, 2014).

Interestingly, research has yet to investigate the effects of the suggested cognitive control advantages for bilinguals on language processing *per se*. In fact, bilinguals are disadvantaged relative to monolinguals on several language-based tasks. For example, relative to monolinguals, bilinguals have smaller vocabularies (Bialystok and Luk, 2012), are slower in picture naming (Gollan et al., 2005; Ivanova and Costa, 2008), show poorer performance on verbal fluency tasks, especially semantic (or category) fluency (Gollan et al., 2002; Bialystok et al., 2008; Luo et al., 2010), and name fewer pictures correctly on standardized naming tests (although this was in L2; Roberts et al., 2002).

However, some aspects of language processing place greater demands on the cognitive control mechanisms that are thought to be boosted in bilinguals. For example, lexical ambiguity processing requires inhibition to suppress the contextually inappropriate meaning of an ambiguous word. The exhaustive access model of lexical ambiguity resolution postulates that all meanings of an ambiguous word are initially activated, followed by rapid selection of the contextually appropriate meaning and suppression/inhibition of all other meanings (Simpson, 1994). Selection of the appropriate meaning has been found to occur in as little as 200 ms (Tanenhaus et al., 1979); however, others have found selective activation of the appropriate meaning of an ambiguous word at less than 150 ms (Simpson, 1981). Another model that has received much empirical support is the reordered access model (Duffy et al., 1988), which hypothesizes an interaction between meaning frequency and context such that meaning activation is exhaustive in order of meaning frequency but context speeds the activation of the intended meaning (e.g., Dopkins et al., 1992; Sereno, 1995; Sheridan et al., 2009).

Early research has demonstrated that both meaning frequency and context contribute to lexical access when an ambiguous word is encountered. Simpson and Burgess (1985) used word pairs comprised of a homograph prime and target words related to either the subordinate or dominant meaning of the prime, or unrelated to either meaning, in a lexical decision task to examine meaning activation. Their findings suggested that initially only the dominant meaning was activated but after 300 ms both meanings were simultaneously active. However, at a longer stimulus onset asynchrony (SOA; i.e., 750 ms) meaning activation was once again restricted to the dominant meaning. Another study used event-related brain potentials (ERPs) to show that following a sentence context and at a short SOA (i.e., 200 ms) activation of the contextually inappropriate meaning of an ambiguous word was delayed relative to activation of the contextually appropriate meaning (Van Petten and Kutas, 1987). However, at a long SOA (i.e., 700 ms) only the contextually appropriate meaning of the homograph was activated.

More recent research has highlighted the importance of cognitive control processes for the resolution of lexical ambiguity. In one study, functional magnetic resonance imaging revealed increased bilateral activation in the inferior frontal gyrus (IFG) in response to prime-target pairs that included an ambiguous word relative to pairs that did not (Bilenko et al., 2009). Given that the IFG has been found to be important for attention and cognitive control (e.g., Miller, 2000; Hampshire et al., 2010) the findings of Bilenko et al. suggest that cognitive control processes are implicated in the processing of ambiguity. Furthermore, Bilenko et al. used a lexical decision task, which does not require the participant to explicitly analyze the semantic content of the stimuli; thus, the findings demonstrate the involvement of cognitive control in ambiguity processing in an implicit task. In another series of studies, children were compared to adults in order to determine how developmental changes in executive functioning impact ambiguity resolution (Khanna and Boland, 2009). Khanna and Boland showed that superior executive function processes were associated with better use of context to resolve ambiguity.

Other studies have found that individuals with impairments in executive functioning (i.e., patients with Alzheimer's disease) show difficulties with ambiguity resolution (e.g., Faust et al., 1997; Balota et al., 2001). Faust et al. examined the level of activation of the contextually inappropriate meaning of ambiguous words using a context verification task. Participants with Alzheimer's disease showed greater interference from the contextually inappropriate meaning of the ambiguous word relative to healthy older adults. In a second experiment Faust et al. demonstrated that participants with Alzheimer's disease were able to use context appropriately to enhance contextually appropriate information, indicating that findings of increased interference from contextually inappropriate material was not the result of a complete inability to use context. Rather, the authors suggested that interference from the inappropriate meaning of the ambiguous word resulted from declines in inhibitory control.

More recently, Hussey and Novick (2012) found that training executive control processes results in improved syntactic ambiguity resolution and suggest that the same may be possible for lexical ambiguity processing. Given that bilingualism is believed to be beneficial for executive control processes, it follows that bilingualism might also exert an influence on lexical ambiguity processing within a language.

In the current study, we investigated whether being bilingual affects the processing of within-language lexical ambiguity. The goal was to determine whether being bilingual affects aspects of language processing *per se* that rely on the cognitive control processes thought to be enhanced in bilinguals, such as lexical ambiguity processing. We examined the effect of bilingualism on the processing of intralingual homonyms (i.e., words with two unrelated meanings within a single language, e.g., *bank*, meaning “a financial institution” or “the edge of a river”) in young adults, using both behavioral and electrophysiological measures. Specifically, we investigated which meaning(s) of a homonym were activated following a biasing sentence context, how this changed over time, and whether this differed for monolinguals and bilinguals. We used a semantic priming paradigm where participants were presented with sentences that terminated in a homonym (e.g., “*She made a deposit at the* *bank*.”) and were required to make a relatedness judgment for target words that were presented following each sentence.

Behaviorally, semantic priming is evidenced by shorter RTs for target words that are preceded by a related word (e.g., *bank* followed by *money*) relative to target words preceded by an unrelated word (e.g., *bank* followed by *shoe*). By varying the context of the sentence and which meaning of the homonym the target word was related to, we were able to determine which meaning(s) of the homonym were activated. For example, given the sentence “*She made a deposit at the* *bank*,” three types of targets were possible, one related to the contextually appropriate meaning of the homonym (e.g., *money*), one related to the contextually inappropriate meaning of the homonym (e.g., *river*), and one unrelated to either meaning of the homonym (e.g., *shoe*). If semantic priming occurred for both related target words, we can infer that both meanings of the homonym were activated.

Semantic priming can also be measured electrophysiologically using ERPs. ERPs are extracted from the ongoing electroencephalogram (EEG), and their amplitude and latency are believed to reflect the strength and timing of the underlying cognitive processes (Coles and Rugg, 1995). ERPs are excellent for measuring language processes because they have exquisite temporal resolution, on the order of milliseconds, and measure cognitive processes as they unfold in time. The ERP component of interest in this investigation is the N400, a negative-going deflection in the waveform that occurs approximately 400 ms following a stimulus; the amplitude of the N400 is inversely related to the expectedness of a target word (Kutas and Hillyard, 1984). That is, an unexpected or unprimed target elicits a larger amplitude N400 relative to an expected or primed target. In the current paradigm, we would expect a smaller amplitude N400 for target words related to the activated meaning(s) of the homonym relative to unrelated targets.

The sensitivity of the N400 to semantic manipulations and its temporal resolution have been capitalized on in previous investigations of lexical ambiguity. For example, Swaab et al. (2003) examined the N400 to target words following the presentation of sentences terminating with a homonym and found initial partial activation of both meanings of the homonym and that the dominant meaning was always partially activated. In another series of studies, Gunter et al. (2003) examined the N400 to determine whether lexical ambiguity resolution relies on activation or inhibition. Their findings demonstrated differences in lexical ambiguity processing between individuals with high and low working memory and suggest that inhibition is particularly important for lexical ambiguity resolution in individuals with high working memory. In a final example, Elston-Güttler and Friederici (2005) examined the processing of different types of homonyms (i.e., both meanings were nouns or one meaning was a noun and the other was a verb) in sentence context for native and non-native speakers of English. They found that at early stages of processing, in general both groups showed similar lexical ambiguity processing, whereas differences between native and non-native speakers were obvious at later stages of processing.

In the current investigation, we hypothesized that, if bilingualism impacts the inhibitory control processes that underlie lexical ambiguity resolution, then we should see differences between monolinguals and bilinguals in the activation of the contextually inappropriate meaning of the homonym. According to the bilingual advantage hypothesis, bilinguals demonstrate superior inhibitory control functions relative to monolinguals; therefore, we predict greater inhibition of the contextually inappropriate meaning of the homonym in bilinguals relative to monolinguals (i.e., longer RTs and larger amplitude N400s for contextually inappropriate targets than contextually appropriate and unrelated targets). Given that both meanings of a homonym are thought to be initially activated, followed by rapid selection of the contextually appropriate meaning, we included a manipulation of the interstimulus interval (ISI) between the sentence-final homonym and the target word in order to examine any language group differences in the timecourse of meaning activation. If monolinguals and bilinguals differ in how they resolve lexical ambiguity and bilinguals use inhibition to a greater extent than monolinguals we would expect to see similar performance from both groups at the short ISI, and differences should emerge at the long ISI. To our knowledge, this is the first study to examine language group differences in within-language ambiguity resolution using both behavioral and electrophysiological measures.

## Methods

### Participants

Twenty-two monolingual and 23 bilingual young adults were tested, but one monolingual and two bilinguals were excluded due to poor EEG recordings, two bilinguals were excluded due to poor second language (L2) proficiency and two bilinguals did not complete the full battery of tasks. Thus, the final sample was comprised of 21 monolingual and 17 bilingual right-handed young adults matched on age and education. Monolinguals were native English speakers with no knowledge of or experience in a second language (seven monolingual participants had minimal exposure to French). Bilinguals were highly proficient in English and French (seven participants reported French to be their native language^1^), having learned their second language before the age of 6 years. Proficiency was measured using self-report and an animacy judgment task (dscribed below; Segalowitz and Frenkiel-Fishman, 2005). All participants demonstrated normal cognitive functioning as measured by the Montreal Cognitive Assessment (MoCA; Nasreddine et al., 2005), and self-reported good health with no medical conditions or medications known to affect cognition. The two groups were matched on age, education, general cognitive function, and working memory (as measured by the Digit Span subtest of the Wechsler Adult Intelligence Scale III; Wechsler, 1997). Participant characteristics can be found in Table 1. Participants were compensated $10 per hour of participation. This study was approved by the Research Ethics Board at the Bruyère Research Institute and the University of Ottawa.

**Table 1:** **Demographic information and participant characteristics for the monolingual and bilingual samples**.

		**Monolinguals (*n* = 21; 10 males)**	**Bilinguals (*n* = 17; 8 males)**
		**Mean *(SD)***	**Mean *(SD)***
Age		21.7 *(1.8)*	20.1 *(1.7)*
Education		15.6 *(1.0)*	15.5 *(1.4)*
MoCA^a^		28.8 *(1.2)*	28.0 *(1.7)*
Digit Span^b^	Forward	10.9 *(1.6)*	11.1 *(1.7)*
	Backward	6.9 *(2.1)*	6.9 *(1.8)*
L1 self-reported language proficiency^c^	Listening	5.0 *(0.0)*	5.0 *(0.0)*
	Reading	5.0 *(0.0)*	4.9 *(0.2)*
	Speaking	5.0 *(0.0)*	5.0 *(0.0)*
	Writing	5.0 *(0.0)*	4.7 *(0.4)*
L2 self-reported language proficiency^c^	Listening	1.6 *(0.8)*	4.7 *(0.6)*
	Reading	1.4 *(0.5)*	4.4 *(0.9)*
	Speaking	1.3 *(0.5)*	4.3 *(1.0)*
	Writing	1.1 *(0.4)*	4.0 *(1.0)*
Coefficient of variability L1		0.18 *(0.05)*	0.24 *(0.08)*
Coefficient of variability L2		n/a	0.24 *(0.06)*

Language groups were matched for age, education, general cognitive function and working memory.

a Maximum score of 30; where a score of 26 or above indicates normal cognitive function.

b Maximum score of 16 for forward and backward digit span.

c 5-point likert scale; 1 = no ability at all, 5 = native-like ability.

### Materials and apparatus

Participants completed several tasks as part of the current experiment, including the MoCA (Nasreddine et al., 2005), an animacy judgment task (Segalowitz and Frenkiel-Fishman, 2005), a vocal Stroop color-word task, and the experimental lexical ambiguity task during which the EEG was recorded.

#### MoCA

The MoCA (Nasreddine et al., 2005) is a 10-min cognitive screening tool designed to detect mild cognitive impairment in older adults; it was included here so that the current data may later be compared to those from an older adult sample. The MoCA is scored on a 30-point scale, with a score of 26 or higher considered normal. It tests several cognitive domains including visuospatial and executive control, naming ability, memory, attention, language, abstraction, and orientation.

#### Animacy judgment task

The animacy judgment task (Segalowitz and Frenkiel-Fishman, 2005) was used as an objective measure of relative L2 proficiency. The task comprises 72 nouns each in English and French, which includes eight practice trials and 64 experimental trials. Stimuli were presented in separate language blocks using E-Prime 2.0 presentation software (Psychology Software Tools, Pittsburg, PA, USA) on a Dell Inspiron mini 1011 laptop with a 10.1 inch screen, an Intel Atom processor, and running Microsoft Windows XP Home Edition. Participants were required to decide as quickly and accurately as possible whether each noun represented a living or non-living object. From these data the coefficient of variability (CV), a measure of intraindividual variability in response time (RT), was calculated separately for each language by dividing each individual's standard deviation for correct trials by their mean RT for correct trials. The CV reflects automaticity of processing; thus, the more similar the CVs in a participant's native language (L1) and L2, the more equally proficient in the two languages the participant was considered to be (see Segalowitz and Segalowitz, 1993).

#### Vocal stroop task

The vocal Stroop task was used as a measure of inhibitory control. Given previous findings demonstrating language group differences in inhibitory control, it was important to determine whether our monolinguals and bilinguals demonstrated this pattern. The task comprises two parts, one blocked and one intermixed. For both tasks stimuli were presented using E-Prime 2.0 presentation software (Psychology Software Tools, Pittsburg, PA, USA) on a Dell OptiPlex 780 desktop computer with Windows XP Professional operating system, an Intel Core 2 Duo processor and a 20″ monitor. Responses were recorded using the E-Prime serial response box and an Audio-Technica Cardioid low impedance microphone.

In the blocked design there were four blocks comprised of 60 trials each: word reading, color naming, congruent color naming, and incongruent color naming, presented in this order. The word reading, congruent color naming, and incongruent color naming conditions comprised the color words *RED, GREEN, YELLOW*, and *BLUE* printed in 24-point Arial font on a black background, whereas the color naming condition comprised circles measuring 5 cm in diameter presented in red, green, yellow, or blue at the center of the monitor on a black background. For the word reading condition the color words were presented in white on a black background and participants were required to read the word as quickly and accurately as possible. In the color naming, congruent color naming and incongruent color naming conditions participants were required to identify the color of the stimulus. In the congruent color naming condition the color word and the color of the font were congruent (e.g., the word *RED* printed in red), whereas in the incongruent color naming condition they were incongruent (e.g., the word *RED* printed in yellow) and participants were required to inhibit the dominant word reading response in order to correctly identify the color of the print.

The intermixed design comprises 144 trials equally distributed among three conditions (i.e., neutral, congruent color naming, and incongruent color naming) and was presented in an intermixed fashion. The congruent and incongruent color naming conditions were identical to those in the blocked design. The neutral condition was comprised of strings of “X”s presented in red, green, yellow, or blue font, with the number of “X”s corresponding to the number of letters in the color word (e.g., *XXX* printed in red). Participants were required to identify the color of the stimulus as quickly and accurately as possible.

#### Lexical ambiguity task

The lexical ambiguity task is of primary interest in the current investigation. In this task participants were presented with sentences terminating in an ambiguous word (i.e., a homonym) and followed by a target word for which a relatedness judgment was required. A typical trial proceeded as follows: the sentence was presented with the final ambiguous word missing (e.g., *The doctor asked her to step onto the* _______.) which stayed on the screen until the participant pressed the spacebar to indicate that they had finished reading the sentence. The homonym then appeared on the screen for 180 ms (e.g., *scale*), and was followed by a target word presented in capital letters (e.g., *BALANCE*) which stayed on the screen until the participant indicated whether it was related to the final word of the sentence or not. The sentences were designed to bias the reading of the sentence terminal homonym toward either its dominant or subordinate meaning; for example, *The doctor asked her to step onto the* _______ vs. *He had trouble completely removing the fish's* _______, where the final word was *scale* for both sentences, the former biasing the dominant meaning and the latter biasing the subordinate meaning. Meaning dominance was determined using the Twilley et al. (1994) relative meaning norms such that the mean proportion of responses endorsing the dominant meaning was 0.74 (*SD* = 0.13) and the mean proportion of responses endorsing the subordinate meaning was 0.15 (*SD* = 0.10). Sentences were between 7 and 11 words in length with a mean length of 8.55 words (*SD* = 1.1) for dominant biasing sentences and a mean length of 8.68 words (*SD* = 1.1) for subordinate biasing sentences, and the sentence length did not differ for the two conditions [*t*_(150)_ = −1.09, *p* = 0.28]. Cloze probability norms were collected from 10 young adults and the dominant (*M* = 0.34, *SD* = 0.32) and subordinate (*M* = 0.34, *SD* = 0.35) conditions were matched for cloze probability of the sentence terminal homonym [*t*_(298)_ = 0.0, *p* = 1]. Target words could be related to either the dominant or the subordinate meaning of the homonym or unrelated to either meaning, creating six experimental conditions; see Table 2 for experimental conditions and sample stimuli. The full list of sentence terminal homonyms and target words can be found in Supplementary Material. In addition, we manipulated the interstimulus interval (ISI) between the terminal homonym and the target word in order to examine the timecourse of meaning activation. We included an immediate condition (0 ms ISI) and a delayed condition (1000 ms) with the same stimuli being seen in each condition. This resulted in a total of 12 experimental conditions.

**Table 2:** **Experimental conditions and sample stimuli**.

**Context**	**Target**		
	**Dominant**	**Subordinate**	**Unrelated**
Dominant: *The doctor asked her to step onto the scale.*	*BALANCE*	*SKIN*	*SHOE*
Subordinate: *He had trouble completely removing the fish's scale.*	*BALANCE*	*SKIN*	*SHOE*

Each sentence was seen six times over the course of the experiment, once followed by each target type in each ISI condition. Target words related to the dominant or subordinate meaning of the homonym were seen four times throughout the experiment, once following each type of biasing context and in each ISI condition. Unrelated target words were seen twice throughout the experiment, once in each ISI condition. Target words were matched across conditions for number of letters, frequency of occurrence (Kučera and Francis, 1967), concreteness, imageability, and familiarity (MRC Psycholinguistic Database, http://websites.psychology.uwa.edu.au/school/MRCDatabase/uwa_mrc.htm).

One hundred and fifty homonyms were selected and divided into three equivalent lists; each participant completed one of the three lists. There were 50 sentences per condition in each list, for a total of 600 sentences in each version of the experiment. Given the number of stimuli, we divided each list into two testing sessions such that participants saw 300 sentences per session. We created the two sessions so that each related target word was seen only twice (once following each biasing context) and each unrelated target word only once during each session. The first and second halves of each list were completed in counterbalanced order. Stimuli were presented in random order using E-Prime 2.0 (Psychology Software Tools, Pittsburg, PA, USA) on a Dell OptiPlex 780 desktop computer with Windows XP Professional operating system, an Intel Core 2 Duo processor and a 20″ monitor.

#### EEG recording

The continuous EEG was recorded from 32 tin electrodes embedded in a commercially available nylon cap (Electro-Cap International, INC., Eaton, OH, USA) positioned according to the international 10–20 system of electrode placement. All active electrode sites were referenced online to linked ears and a cephalic site was used as the ground. The horizontal and vertical electro-oculogram was recorded from electrodes placed at the outer canthus of each eye and from electrodes placed above and below the left eye, respectively. The EEG was amplified using NeuroScan NuAmps (NeuroScan, El Paso, TX, USA) and was sampled at a rate of 500 Hz in a DC to 100 Hz bandwidth. EEG data were processed offline using NeuroScan 4.5 EDIT software (NeuroScan, El Paso, TX, USA). Offline data processing consisted of applying a 30 Hz lowpass filter, correcting vertical EOG artifact using a spatial filter (NeuroScan EDIT 4.3) and excluding trials containing deflections exceeding ±100 μV as well as those where horizontal EOG artifact exceeded ±50 μV. The electrophysiological time epoch was 1100 ms, comprising a 100 ms pre-stimulus baseline and 1000 ms following the onset of the target word. Averages were computed based on the 12 conditions of the experimental task and were baseline corrected to a 0 μV average of the 100 ms pre-stimulus interval.

### Procedure

Participants visited the laboratory on two separate occasions separated by approximately 1 week, each lasting 1.5–2.0 h. During the first visit, written informed consent was obtained, and the MoCA, animacy judgment task, and vocal Stroop task were administered provided that they had not previously been completed by the participant as part of any previous studies in the laboratory. Following this, participants were set up with the EEG cap and one half of the experimental lexical ambiguity task was completed while EEG recording took place. During the second visit, the other half of the experimental task was completed while EEG recording took place.

At the end of the second testing session participants were debriefed and any questions that they had were answered.

## Results

All statistical analyses were conducted using PASW Statistics 18 using an alpha level of 0.05, unless otherwise specified. Trials for which RTs were greater than ±2.5 standard deviations from the mean were excluded from the behavioral data as outliers.

### Animacy judgment task

In bilinguals the CV was calculated separately for each individual in each language by dividing their *SD* for correct trials by their mean RT for correct trials. There was a significant Pearson Correlation between the CV in L1 and L2 (*r* = 0.73, *p* < 0.01) and a paired samples *t*-test indicated that there was no significant difference between the two CVs [*t*_(16)_ = −0.66, *p* = 0.5] suggesting high relative proficiency in L2.

### Vocal stroop task

Raw data are presented in Table 3. Due to microphone error, three monolinguals and two bilinguals were excluded from analysis of the Stroop task. A mixed-design analysis of variance (ANOVA) was conducted separately for the blocked and intermixed versions of the vocal Stroop task with the between subjects variable Language Group (monolingual vs. bilingual) and the within subjects variable Condition (blocked design: word reading, color naming, congruent color naming, and incongruent color naming; intermixed design: neutral, congruent, incongruent). Both ANOVAs revealed no significant effect of Language Group [blocked: *F*_(1, 31)_ = 0.93, *p* = 0.3; intermixed: *F*_(1, 31)_ = 2.4, *p* = 0.1] or Language Group x Condition interaction [blocked: *F*_(3, 93)_ = 0.88, *p* = 0.4; intermixed: *F*_(2, 62)_ = 0.57, *p* = 0.6]; such an interaction would have been expected if there was an effect of bilingualism on inhibitory control (as measured by the Stroop task) in this sample. There was, however, a main effect of Condition for both versions of the tasks, indicating that the task was in fact introducing interference. Specifically, in the blocked design, RTs for the incongruent color naming condition were longer than for the three other conditions, and word reading was faster than the other three conditions, while color naming and congruent color naming did not differ [*F*_(3, 93)_ = 91.9, *MSE* = 5720.7, *p* < 0.001]. For the intermixed design, all three conditions differed from each other, with neutral trials eliciting the fastest responses and incongruent trials eliciting the longest [*F*_(2, 62)_ = 102.5, *MSE* = 1684.5, *p* < 0.001].

**Table 3:** **Data from the vocal Stroop Task**.

		**Monolinguals**		**Bilinguals**	
		**RT**	**Accuracy**	**RT**	**Accuracy**
		**Mean *(SD)***	**Mean *(SD)***	**Mean *(SD)***	**Mean *(SD)***
Blocked	Word reading	444.2 *(50.5)*	97.8 *(3.4)*	467.0 *(77.2)*	97.5 *(5.8)*
	color naming	531.8 *(58.6)*	98.4 *(2.4)*	557.0 *(59.1)*	96.8 *(3.8)*
	Congruent color naming	517.3 *(94.1)*	71.4 *(2.7)*	506.3 *(122.6)*	71.2 *(4.2)*
	Incongruent color naming	721.9 *(141.8)*	93.9 *(9.0)*	771.2 *(86.9)*	95.8 *(4.0)*
Intermixed	Congruent	668.8 *(96.0)*	71.6 *(2.8)*	707.5 *(88.9)*	73.5 *(2.2)*
	Incongruent	768.1 *(129.4)*	92.4 *(6.7)*	826.8 *(97.5)*	94.4 *(5.0)*
	Neutral	632.0 *(99.9)*	71.9 *(3.3)*	687.9 *(78.4)*	71.9 *(3.0)*

In addition to analysing the raw RTs, we also examined Stroop interference (i.e., the increase in RT for incongruent trials relative to congruent or neutral trials). In total we calculated five different Stroop interference values by subtracting the RT for each of the congruent and neutral conditions in the blocked and intermixed designs from the respective incongruent condition. We conducted a oneway ANOVA on each interference effect to compare monolinguals and bilinguals; there were no significant effects of Language Group (all *p*s > 0.2).

### Lexical ambiguity task

#### Behavioral results

**Figure 1:**
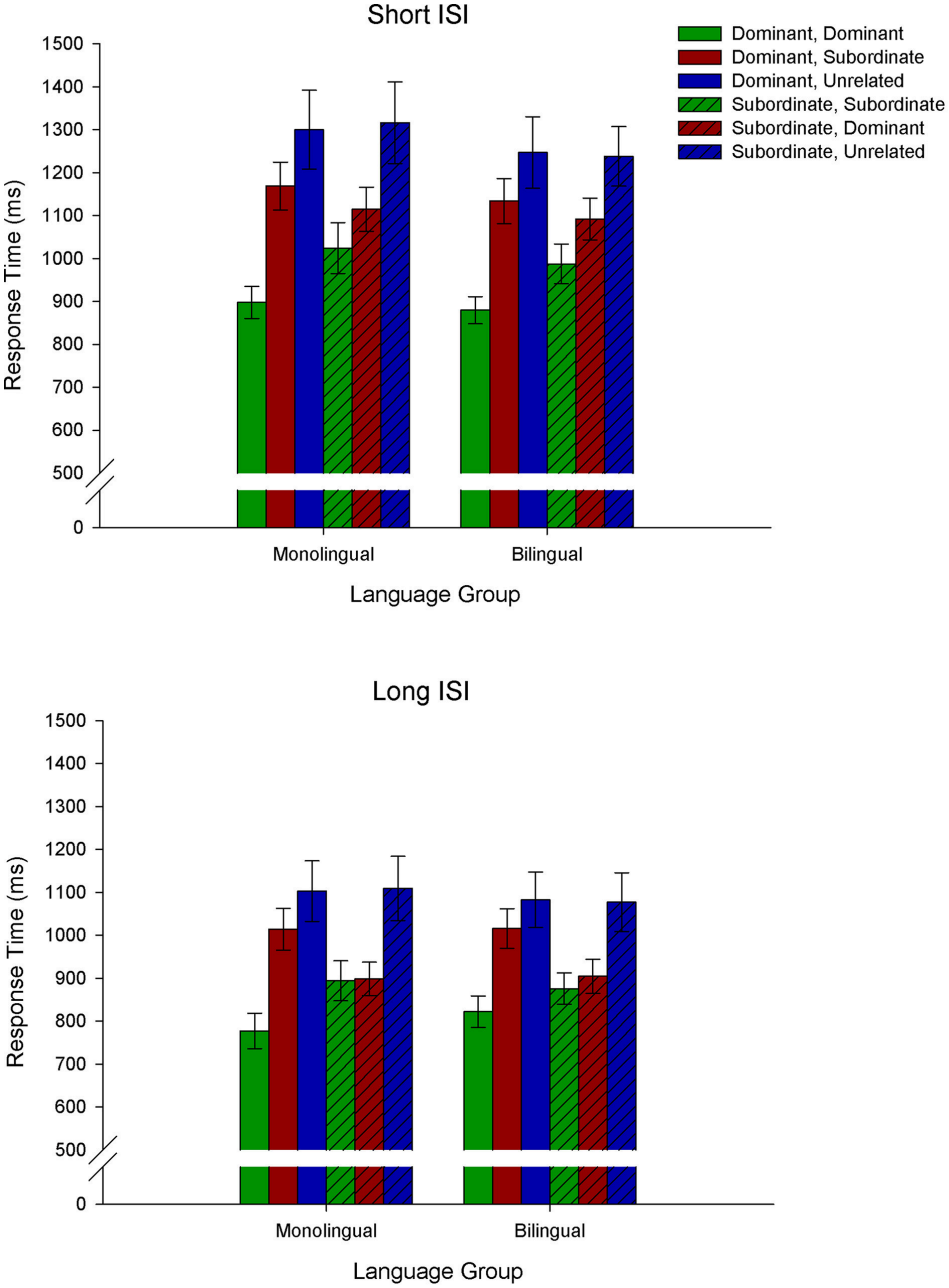
**Behavioral results demonstrating response time as a function of Context × Target type (e.g., *Dominant, Dominant* refers to a dominant biasing context followed by a target related to the dominant meaning of the homonym; *Dominant, Subordinate* refers to a dominant biasing context followed by a target related to the subordinate meaning of the homonym, and *Dominant, Unrelated* refers to a dominant biasing context followed by a target unrelated to either meaning of the homonym)**.

The RT data are depicted in Figure 1. We conducted a 2 (Language Group: monolingual, bilingual) × 2 (Context: dominant, subordinate) × 3 (Target: dominant, subordinate, unrelated) × 2 (ISI: short, long) repeated measures ANOVA on the RT data. Significant interactions were decomposed with Bonferroni corrected simple effects analyses.

There was a main effect of Target, *F*_(2, 72)_ = 35.4, *MSE* = 74, 473.3, *p* < 0.001, demonstrating that all three target types differed from each other, with the fastest RTs for targets related to the dominant meaning of the homonym and the longest to targets unrelated to either meaning. There was also a main effect of ISI, *F*_(1, 36)_ = 124.4, *MSE* = 20, 978.9, *p* < 0.001, demonstrating that responses were faster at the long ISI relative to the short ISI. In addition, there was a Context × Target × ISI interaction, *F*_(2, 72)_ = 10.7, *MSE* = 4230.5, *p* < 0.001, showing that at both the short and long ISIs, responses differed for the three target types following a dominant biasing context: targets related to the dominant meaning of the homonym elicited the fastest RTs, and unrelated targets the longest. However, RTs in response to targets following a subordinate biasing context differed for the short and long ISIs. Specifically, at the short ISI all three target types differed, with targets related to the subordinate meaning of the homonym eliciting the fastest RTs and unrelated targets the longest. At the long ISI, in contrast, targets related to the dominant and subordinate meaning of the homonym did not differ from each other, but elicited faster RTs than unrelated targets. Of importance is that there was no main effect of Language Group [*F*_(1, 36)_ = 0.09, *p* = 0.8] nor significant interactions with Language Group (all *p*s > 0.2), indicating similar performance for monolinguals and bilinguals.

Accuracy data were difficult to interpret and were therefore analyzed primarily to ensure that participants were in fact performing the task. That is, given that participants were asked to indicate whether a target word was related to an ambiguous sentence terminal word, in cases where the target was related to the contextually inappropriate meaning both a “yes” or “no” response could be taken as a correct response. However, accuracy in the contextually appropriate and unrelated conditions was used to ensure that participants were attending to the stimuli. All participants showed accuracy rates above 75% for the appropriate and unrelated conditions (Monolinguals—short ISI appropriate: *M* = 95.3, *SD* = 4.3; short ISI unrelated: *M* = 89.9, *SD* = 5.7; long ISI appropriate: *M* = 92.1, *SD* = 4.6; long ISI unrelated: *M* = 94.1, *SD* = 3.5; Bilinguals—short ISI appropriate: *M* = 92.3, *SD* = 6.6; short ISI unrelated: *M* = 91.7, *SD* = 5.2; long ISI appropriate: *M* = 90.8, *SD* = 6.5; long ISI unrelated: *M* = 92.3, *SD* = 5.0). Monolinguals and bilinguals did not differ in accuracy (all *p*s > 0.1).

#### Electrophysiological results

**Figure 2:**
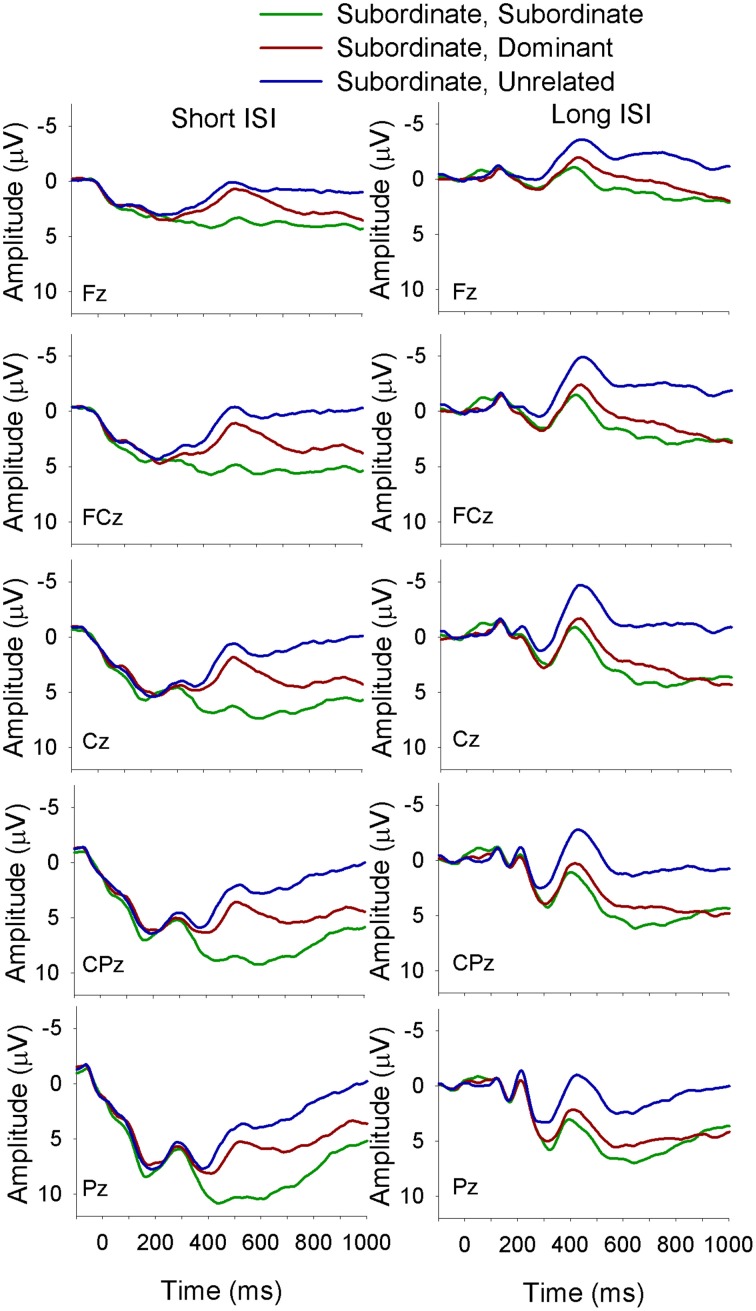
**ERP waveforms for monolinguals at midline sites following a subordinate biasing context at the short (left panel) and long (right panel) ISI**. *Subordinate, Subordinate* refers to a subordinate biasing context followed by a target related to the subordinate meaning of the homonym; *subordinate, dominant* refers to a subordinate biasing context followed by a target related to the dominant meaning of the homonym, and *subordinate, unrelated* refers to a subordinate biasing context followed by a target unrelated to either meaning of the homonym.

**Figure 3:**
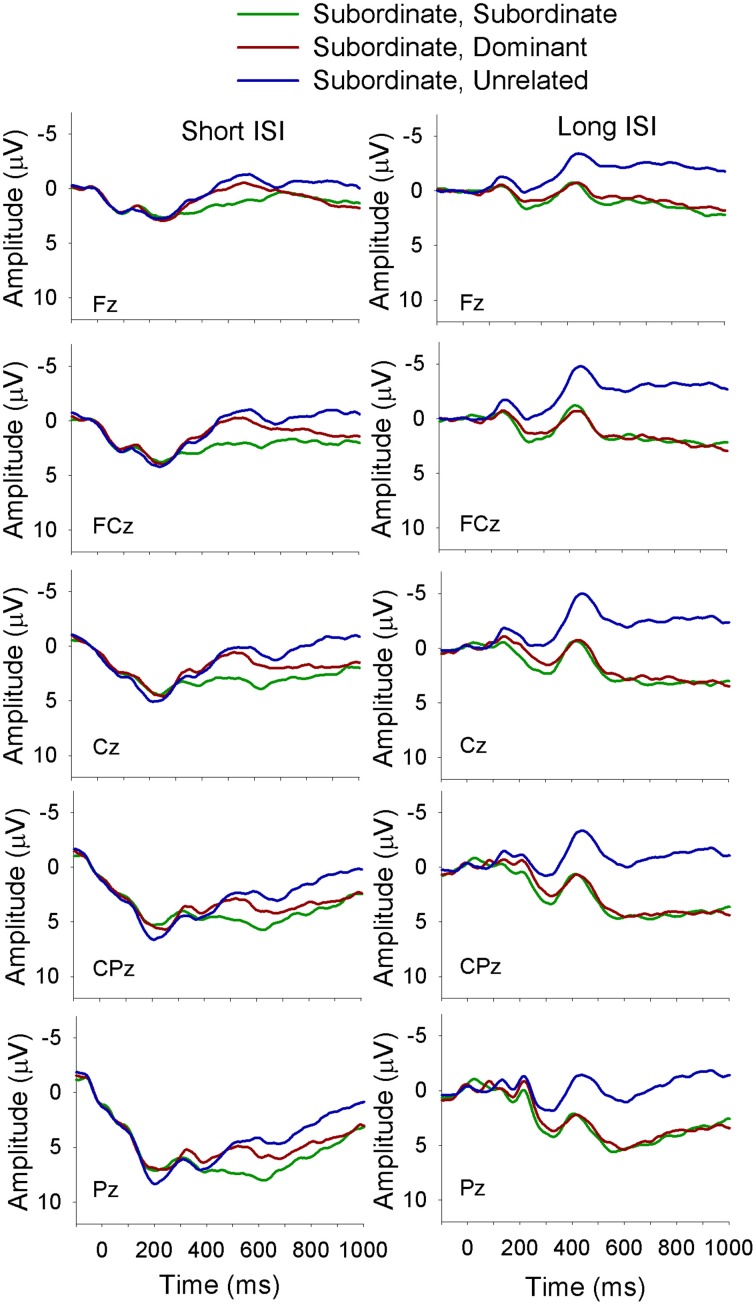
**ERP waveforms for bilinguals at midline sites following a subordinate biasing context at the short (left panel) and long (right panel) ISI**. *Subordinate, Subordinate* refers to a subordinate biasing context followed by a target related to the subordinate meaning of the homonym; *subordinate, dominant* refers to a subordinate biasing context followed by a target related to the dominant meaning of the homonym, and *subordinate, unrelated* refers to a subordinate biasing context followed by a target unrelated to either meaning of the homonym.

**Figure 4:**
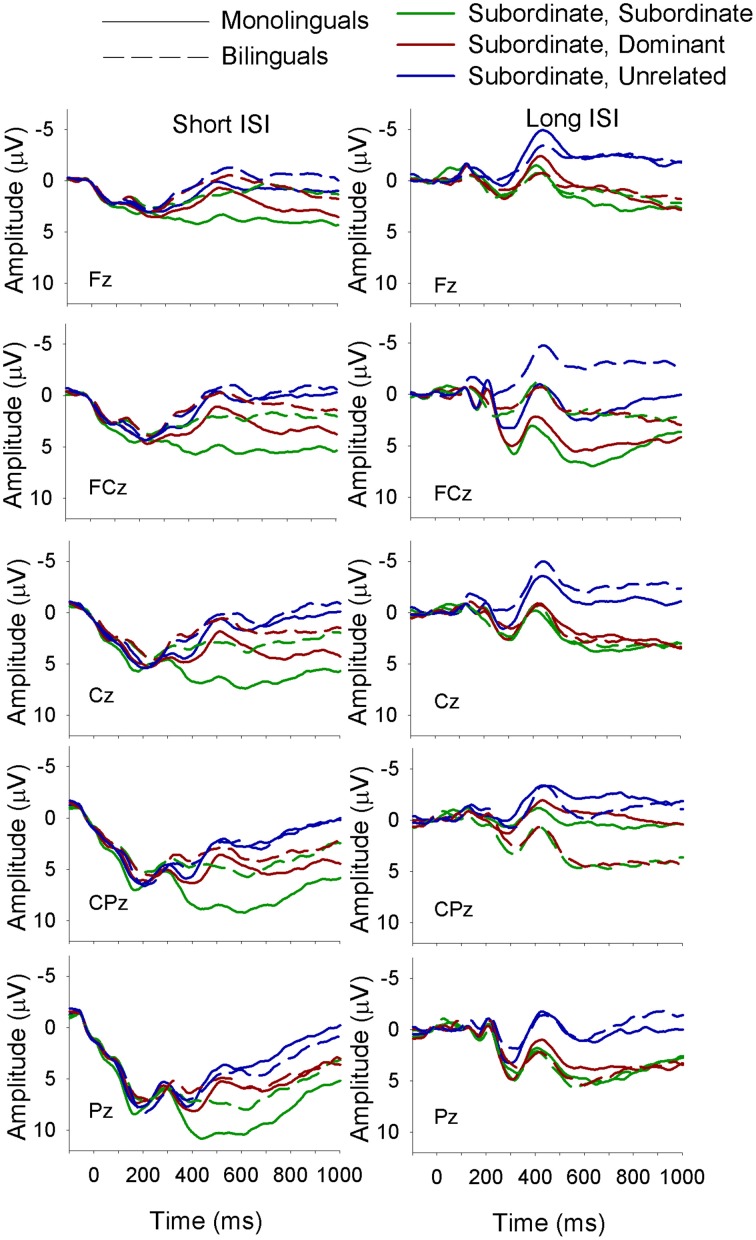
**ERP waveforms for monolinguals and bilinguals at midline sites following a subordinate biasing context at the short (left panel) and long (right panel) ISI**. *Subordinate, Subordinate* refers to a subordinate biasing context followed by a target related to the subordinate meaning of the homonym; *subordinate, dominant* refers to a subordinate biasing context followed by a target related to the dominant meaning of the homonym, and *subordinate, unrelated* refers to a subordinate biasing context followed by a target unrelated to either meaning of the homonym.

**Figure 5:**
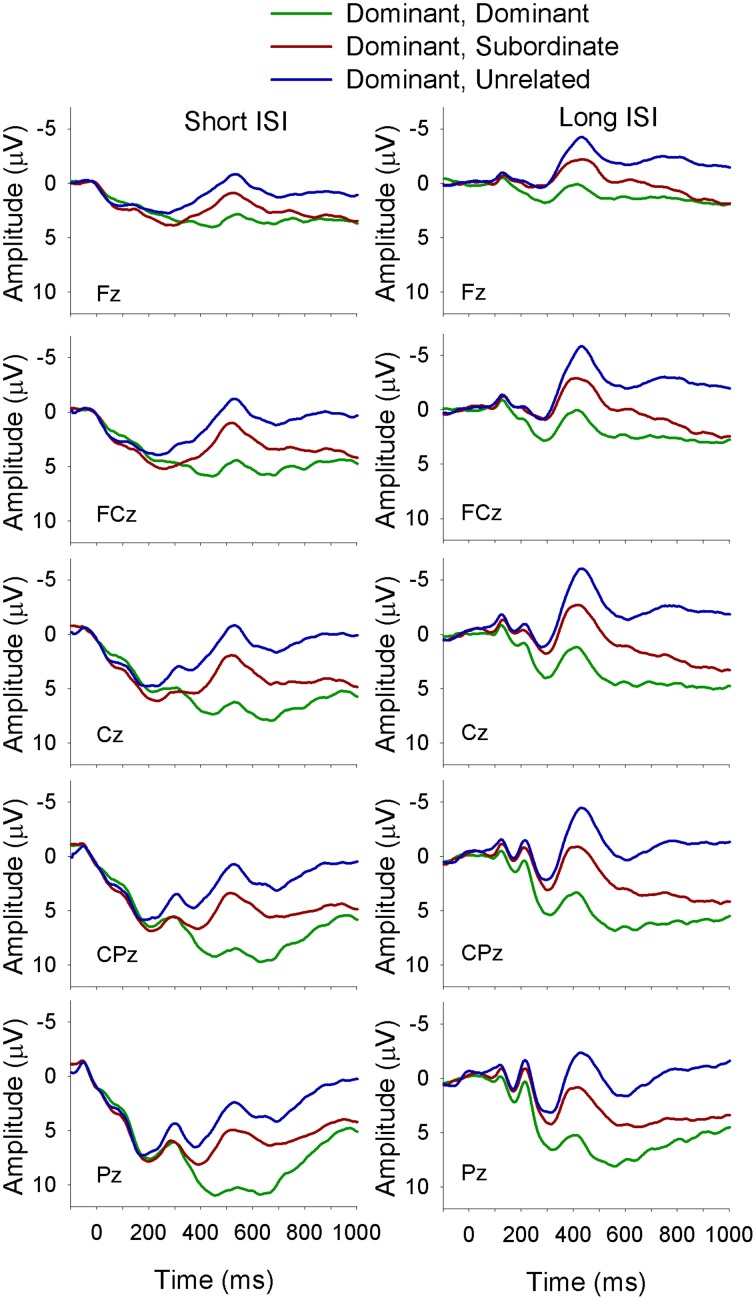
**ERP waveforms for monolinguals at midline sites following a dominant biasing context at the short (left panel) and long (right panel) ISI**. *Dominant, Dominant* refers to a dominant biasing context followed by a target related to the dominant meaning of the homonym; *Dominant, Subordinate* refers to a dominant biasing context followed by a target related to the subordinate meaning of the homonym, and *Dominant, Unrelated* refers to a dominant biasing context followed by a target unrelated to either meaning of the homonym.

**Figure 6:**
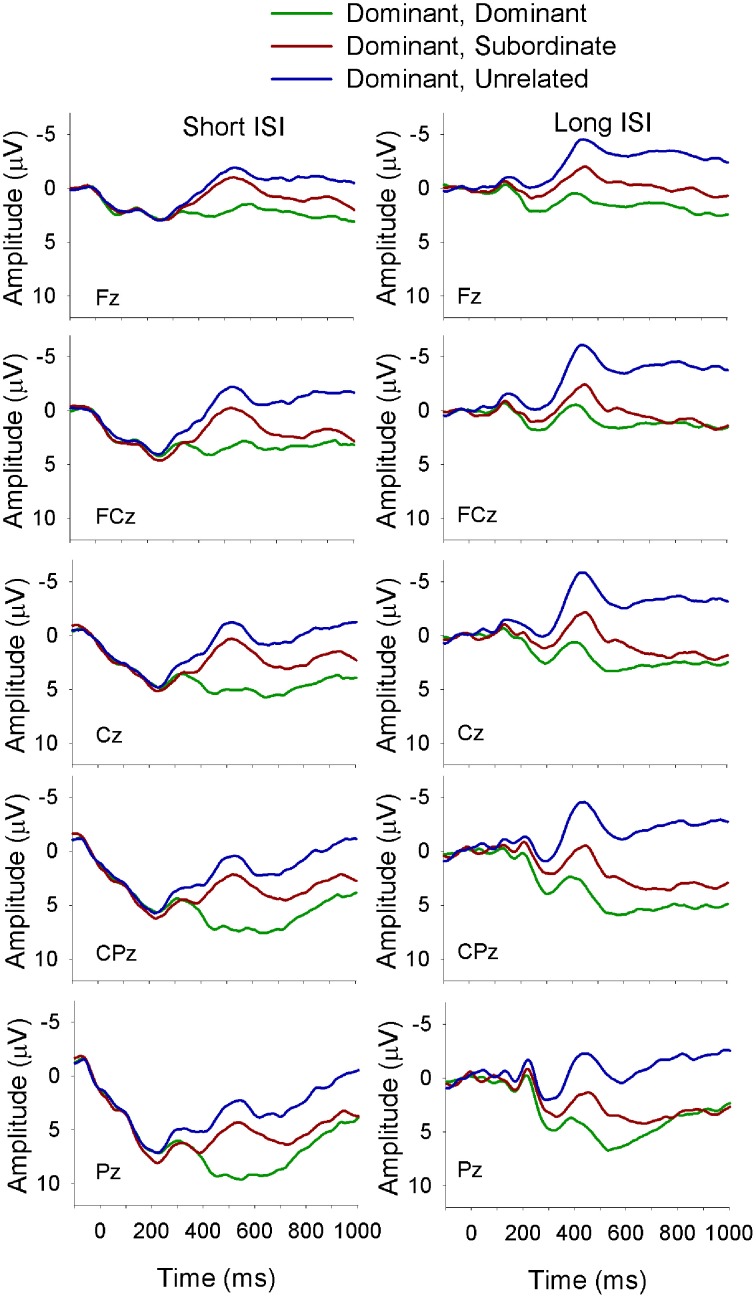
**ERP waveforms for bilinguals at midline sites following a dominant biasing context at the short (left panel) and long (right panel) ISI**. *Dominant, Dominant* refers to a dominant biasing context followed by a target related to the dominant meaning of the homonym; *Dominant, Subordinate* refers to a dominant biasing context followed by a target related to the subordinate meaning of the homonym, and *Dominant, Unrelated* refers to a dominant biasing context followed by a target unrelated to either meaning of the homonym.

**Figure 7:**
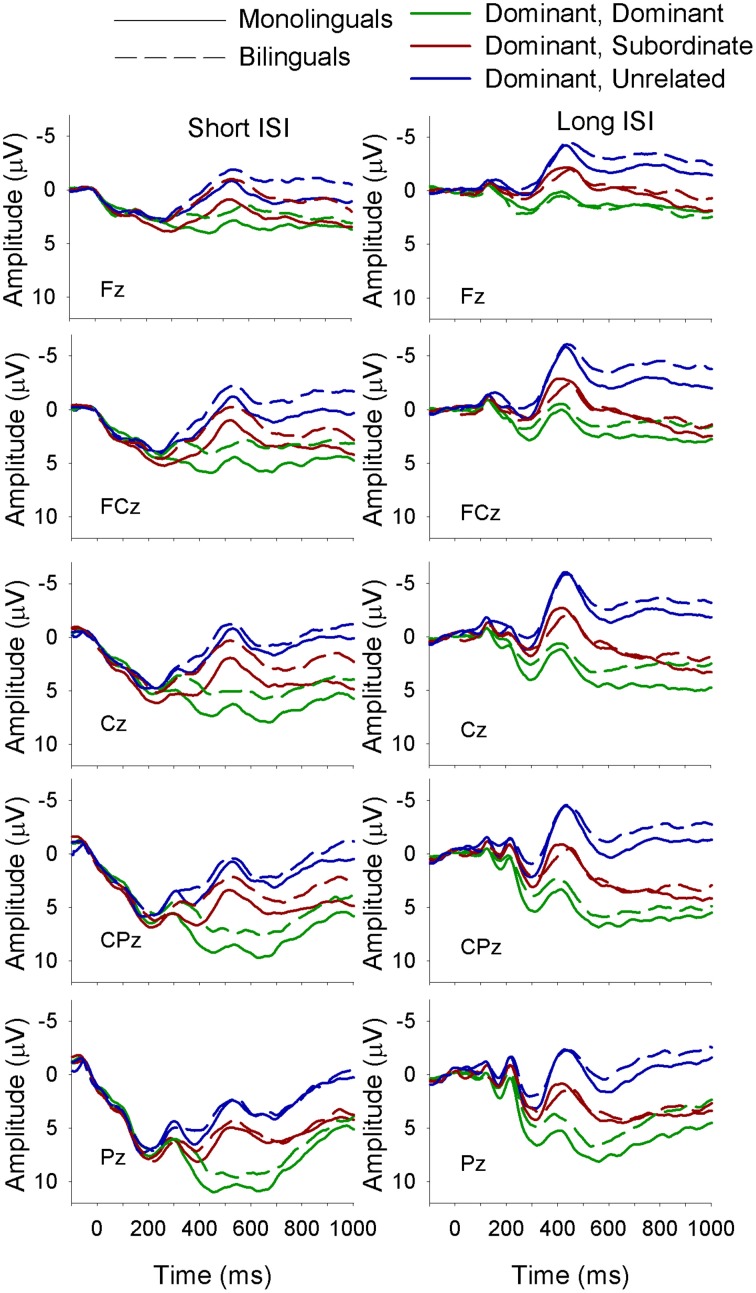
**ERP waveforms for monolinguals and bilinguals at midline sites following a dominant biasing context at the short (left panel) and long (right panel) ISI**. *Dominant, Dominant* refers to a dominant biasing context followed by a target related to the dominant meaning of the homonym; *Dominant, Subordinate* refers to a dominant biasing context followed by a target related to the subordinate meaning of the homonym, and *Dominant, Unrelated* refers to a dominant biasing context followed by a target unrelated to either meaning of the homonym.

The electrophysiological data were analyzed using a mixed-design ANOVA with the between-subjects factor of Language Group (monolingual, bilingual) and the within-subject factors of Context (dominant, subordinate), Target (dominant, subordinate, unrelated), ISI (short, long), site (Fz, FCz, Cz, CPz, Pz)^2^ and Time. The factor Time had six levels that were created by subdividing the time window of interest (i.e., 300–600 ms) into 50 ms time intervals (i.e., 300–350, 350–400 to 550–600 ms). The dependent variable was the mean amplitude of the waveform in each time interval. All significant interactions were decomposed with Bonferroni corrected simple effects analyses. For all analyses with more than one degree of freedom in the numerator the Greenhouse and Geisser (Greenhouse and Geisser, 1959) correction for non-sphericity was applied; following convention we report the unadjusted degrees of freedom, the adjusted mean square error, the adjusted *p*-value, and the Greenhouse-Geisser epsilon value (ε). Figures 2–7 depict the electrophysiological data.

There was a Language Group × Target × Time interaction [*F*_(10, 360)_ = 2.5, *MSE* = 12.5, *p* = 0.03, ε = 0.5] showing that in monolinguals unrelated targets elicited a larger amplitude N400 than targets related to both the dominant and the subordinate meaning of the homonym, which did not differ from each other; this finding held across all six time intervals included in the analysis. The bilinguals showed a similar pattern as the monolinguals from 300 to 400 ms and from 500 to 600 ms, and a divergence in the waveforms resulting in a significant difference between all three target types from 400 to 500 ms, with the largest amplitudes for unrelated targets and smallest for those related to the dominant meaning of the homonym.

A Language Group × Context × Target × Site interaction [*F*_(8, 288)_ = 2.5, *MSE* = 8.0, *p* = 0.04, ε = 0.59] showed that following a dominant biasing context, all three target types differed from each other, with the largest amplitude N400 for unrelated targets and the smallest for targets related to the dominant meaning of the homonym in both language groups at all midline sites. However, following a subordinate biasing context, monolinguals showed differentiation between all three target types, with the largest N400 for unrelated targets and the smallest for targets related to the subordinate meaning at all sites, while bilinguals showed similar amplitude N400 for targets related to both the subordinate and dominant meaning, which were smaller than the N400 elicited by unrelated targets.

Finally, there was a Context × Target × ISI × Site × Time interaction [*F*_(40, 1440)_ = 5.0, *MSE* = 1.5, *p* < 0.01, ε = 0.18]. In short, this complex interaction demonstrated that at the short ISI and following a dominant biasing context all three target types differed from each other (unrelated targets elicited the largest amplitude N400 and targets related to the dominant meaning the smallest). Following a subordinate biasing context a similar pattern emerged (unrelated targets elicited the largest amplitude N400 and targets related to the subordinate meaning the smallest); however, this finding was evident only for the later time intervals, and was not consistent across the electrode sites. At the long ISI and following a dominant biasing context a similar pattern was seen as for the short ISI. On the other hand, at the long ISI and following a subordinate biasing context, unrelated targets elicited a larger amplitude N400 than targets related to both the dominant and subordinate meaning (which did not differ from each other), and this was true for all time intervals at all electrode sites.

In addition, there was a main effect of Target type [*F*_(2, 72)_ = 121.6, *MSE* = 17086.0, *p* < 0.01, ε = 0.91], demonstrating a significant difference in N400 amplitude between all three target types, with the largest amplitude for unrelated targets and the smallest for targets related to the dominant meaning of the homonym. A main effect of ISI was also observed [*F*_(1, 36)_ = 36.3, *MSE* = 913.4, *p* < 0.01], demonstrating larger N400 amplitudes at the long relative to the short ISI. There was no main effect of Language Group [*F*_(1, 36)_ = 1.0, *p* = 0.3].

## Discussion

The purpose of the current investigation was to examine whether being bilingual exerts an effect on aspects of language processing that place demands on cognitive control, specifically lexical ambiguity resolution. Given previous findings suggesting that bilinguals benefit from an advantage in inhibitory control, we expected that these language group differences would carry over into the language domain, with bilinguals demonstrating superior lexical ambiguity resolution relative to their monolingual counterparts. Our results demonstrate that there are subtle differences in how monolinguals and bilinguals process ambiguous words; however, the outcome of the processing does not differ for the two language groups. That is, there were no behavioral differences between the two groups, but there were electrophysiological differences suggesting processing differences—although not necessarily an advantage for bilinguals.

Based on previous research, we hypothesized that initially both meanings of the homonym would be activated, followed quickly by the suppression of the contextually inappropriate meaning. Behaviorally, we expected that this would be evidenced by faster RTs for target words related to both meanings of the homonym relative to unrelated targets at the short ISI, but only for targets related to the contextually appropriate meaning of the homonym at the long ISI. Furthermore, we expected that at the long ISI targets related to the inappropriate meaning of the homonym would elicit RTs that were longer than those in response to unrelated targets, providing evidence for suppression/inhibition of the inappropriate meaning of the homonym. In terms of language group differences, we expected that bilinguals would show stronger and/or more efficient inhibition than monolinguals.

These hypotheses were not supported. Rather, we found that following a dominant biasing context, targets related to the dominant meaning of the homonym (i.e., appropriate targets) elicited the fastest RTs, followed by targets related to the subordinate meaning (i.e., inappropriate targets), followed by unrelated targets, at both the short and the long ISIs in both monolinguals and bilinguals. This indicates that even at the 0 ms ISI, the appropriate meaning of the homonym was selected and activated to a greater extent than the inappropriate meaning, which remained partially activated; there appeared to be no inhibition of the inappropriate meaning. Following a subordinate biasing context we found a similar pattern at the short ISI (contextually appropriate targets elicited the shortest RTs and unrelated the longest); however, at the long ISI both meanings of the homonym were similarly activated, and targets related to both meanings elicited faster RTs than unrelated targets. These findings suggest that the dominant meaning of the homonym was activated regardless of the subordinate biasing context, but this activation occurred following initial activation of the subordinate/appropriate meaning. This finding is not consistent with previous research and suggests selective access of the appropriate meaning of the homonym following a subordinate biasing context at the 0 ms ISI and exhaustive access later in the processing pipeline. Selective activation of the appropriate meaning of the homonym at the short ISI may indicate that the SOA was not short enough to measure differences in the timecourse of meaning activation. Evidence for this comes from previous research that has found selective activation in as little as 150 ms (Simpson, 1981). More recently, Sheridan and Reingold (2012) found that context exerted an influence in as little as 139 ms, and Sereno et al. (2003) used ERPs to show the effect of context on lexical access in the early N1 component (132–192 ms). In the current experiment SOA was 180 ms, the time that the sentence final word remained on the screen.

Critically, the behavioral data do not demonstrate any language group differences. Given that we found no differences between the two groups on the vocal Stroop task, which we took as a basic measure of inhibitory control, this is unsurprising. This absence of an inhibitory control difference between our monolinguals and bilinguals may be explained by Green and Abutalebi's (2013) adaptive control hypothesis. According to the adaptive control hypothesis, language group differences in cognitive control will differ depending on the demands placed on these processes by the language environment and interactional contexts. The participants in this study were from the Ottawa, Canada, region; a highly bilingual area that could be considered by Green and Abutalebi to be a “dense-code-switching context.” Following the adaptive control hypothesis, it would be expected that our bilingual participants from a dense-code-switching context perform no differently than monolinguals.

However, despite a lack of behavioral differences, we did find some language group differences in ambiguity processing in the electrophysiological data. Specifically, we found differences between the two language groups in terms of target type activation over time, and a language group difference in the Context × Target interaction.

With respect to language group differences in target type activation, monolinguals showed greater activation of both meanings of the homonym (i.e., smaller N400 amplitude in response to targets related to both meanings) relative to unrelated targets across the entire N400 window. This was only the case early and late in the time window for bilinguals, who showed less activation of the subordinate meaning of the homonym relative to the dominant meaning during the peak of the N400 (400–500 ms). This finding was irrespective of context; however, there was also a Language Group × Context × Target × Site interaction showing a different pattern of activation for the different target types in relation to context for the monolinguals and bilinguals. Specifically, monolinguals were found to show greater activation of the appropriate meaning of the homonym than the inappropriate meaning (which was activated to a greater extent than unrelated targets) following both a dominant and subordinate biasing context. Bilinguals demonstrated the same pattern following a dominant biasing context, whereas following a subordinate biasing context they showed similar activation of both appropriate and inappropriate meanings.

Taken together these findings suggest that monolinguals process ambiguous words based on the context within which the ambiguous word is encountered, whereas bilinguals only do this following a dominant biasing context; after a subordinate biasing context, in contrast, they activate both meanings equally. One would expect that similar activation of both the contextually appropriate and inappropriate meaning of a word would lead to comprehension difficulties; however, behavioral performance did not differ between the monolinguals and bilinguals, suggesting that the bilinguals were successfully able to manage the activation of multiple meanings in the task used here and respond in a manner similar to their monolingual counterparts. Furthermore, this finding demonstrates that there are subtle differences in how young monolinguals and bilinguals process ambiguity, but the behavioral outcome remains the same, at least in the task used here. This study is not the first to find language group differences in electrophysiological measures in the absence of behavioral differences; for example, Kousaie and Phillips (2012b) report a similar electrophysiological-specific effect from a series of tasks measuring cognitive control.

In addition to language group effects, there were several findings of interest that did not relate to language group and that may have implications for general theories of lexical ambiguity processing. In particular, there was a Context × Target × ISI × Site × Time interaction, showing that following a subordinate biasing context, initially (i.e., at the short ISI) there was greater activation of the subordinate/appropriate than the dominant/inappropriate meaning of the homonym, whereas later (i.e., at the long ISI) both meanings were activated to the same extent. This suggests that there is delayed access to the dominant meaning of the homonym following a subordinate biasing context. However, following a dominant biasing context there was greater activation of the dominant/appropriate meaning of the homonym regardless of ISI. This finding suggests an interaction between meaning frequency and context; that is, following a dominant biasing context, meaning selection is driven by the prior context, whereas following a subordinate biasing context there appears to be initial selective activation of the appropriate meaning, with the higher frequency meaning becoming activated later. Additional support for an interaction between meaning frequency and prior context comes from our behavioral results, which showed faster RTs to appropriate targets than inappropriate targets following a subordinate context at the short ISI, but similar RTs to both appropriate and inappropriate targets following the long ISI; following a dominant biasing context, in contrast, appropriate targets elicited faster RTs for both the short and the long ISIs. Thus, both our behavioral and electrophysiological data support the suggestion that the effect of prior context differs depending on which meaning of an unbalanced homonym it is biasing.

Although we found selective activation of the appropriate meaning of the homonym in some cases, we did not observe any evidence of suppression/inhibition of the inappropriate meaning of the homonym in any of our experimental conditions, behaviorally or electrophysiologically. It is possible that in the current paradigm the inappropriate meaning is not actively inhibited, and a different measure/paradigm may have been better able to detect language group differences. For example, Faust et al. (1997) used a subtraction measure to examine interference from the inappropriate meaning of ambiguous words. They examined RT differences to target words (e.g., *ACE*) following sentences with an ambiguous terminal word (e.g., He dug with the *spade*) and sentences with an unambiguous terminal word (e.g., He dug with the *shovel*) and were able to quantify interference by subtracting RTs following unambiguous terminal words from RTs following ambiguous terminal words.

One aspect of the current design that may be thought to have influenced the results is the repetition of stimuli. Repetition priming refers to the facilitation of the processing of a stimulus on its second presentation. In the current design target words could be seen twice within a testing session, but never following the same biasing context, and four times over the two testing sessions, once following each ISI. That is, target words were seen following a sentence terminal homograph that was biased to each meaning (e.g., the target word *BALANCE* following the sentence *The doctor asked her to step onto the scale* or the sentence *He had trouble completely removing the fish's scale*) within a testing session, and in the subsequent testing session the same sentence-target pair was seen again but with a different ISI (i.e., 0 or 1000 ms). Given the variation in the context of the repetitions it is unlikely that our results are a product of repetition priming. However, Besson and Kutas (1993) demonstrated repetition priming for ambiguous words, even when the ambiguous words were preceded by a different sentence context and when they were biased toward their alternate meaning. In Besson and Kutas' study participants were instructed to read a series of sentences that terminated in an ambiguous word for comprehension, and to try to remember the terminal word in order to complete a cued-recall task. The ambiguous word repetitions were seen in blocks and each block was followed by a cued-recall task in which the sentences were presented with the final word missing and participants were required to complete the sentence. In the current investigation participants were required to make a relatedness judgment in response to a target word following a sentence terminal homograph and responses were measured to the target word, not the ambiguous sentence terminal word. Furthermore, given the random presentation of stimuli and the counterbalancing strategies that we used we are confident that our findings were not influenced by repetition priming. Moreover, if there were effects of repetition priming we would expect them to be similar for the two language groups, therefore not impacting our critical findings.

In sum, we did not find any behavioral evidence for language group differences in lexical ambiguity processing, but the electrophysiological results revealed differences in how monolinguals and bilingual process lexical ambiguity. Specifically, monolinguals appear to rely on context to a greater extent than bilinguals to guide their reading of ambiguous words. Bilinguals, on the other hand, show less selective activation of the contextually appropriate meaning of a homonym than monolinguals. However, there were no behavioral consequences in response to the simultaneous activation of multiple meanings in bilinguals, suggesting that bilinguals are successfully able to manage the activation of multiple meanings and perform similarly to monolinguals in language tasks.

## Author contributions

SK conceived of the study, designed the materials and implemented the experiment. She was also principally responsible for data analysis, interpretation, and manuscript preparation. CL and RLZ contributed invaluably to participant recruitment, data collection, processing and interpretation, and provided input for manuscript preparation. VT supervised this research and contributed to all aspects of the study from study design to manuscript preparation.

## Funding

This research was supported by a Canadian Institutes for Health Research (CIHR) catalyst grant awarded to VT and SK. SK was supported by a CIHR postdoctoral fellowship.

### Conflict of interest statement

The authors declare that the research was conducted in the absence of any commercial or financial relationships that could be construed as a potential conflict of interest.

## Footnotes

^1^Note that the experimental data for the two groups of bilinguals were collapsed for all statistical analyses following a comparison of the two groups using independent samples *t*-tests which demonstrated no systematic differences in performance of the experimental task between those who reported French as their L1 and those who reported English as their L1. This was performed as a precautionary measure despite relatively equal proficiency in both languages in all bilinguals. It is noteworthy that the two bilingual groups also did not differ with respect to age, education, general cognitive function, age of second language acquisition, or second language proficiency.

^2^Four additional analyses that included lateral sites were also conducted, one for each hemisphere and two comparing the two hemispheres (one for each ISI). These analyses yielded similar findings; however, they are not reported here for simplicity and given that the most important and meaningful findings were captured by analysis of the midline sites. The results from these analyses can be found in the Supplementary Material.
