# Supplementary material for: A Behavioral and Electrophysiological Investigation of the Effect of Bilingualism on Lexical Ambiguity Resolution in Young Adults
Source: Front Hum Neurosci. 2015 Dec 21;9:682. doi: 10.3389/fnhum.2015.00682 (PMC4685109; doi:10.3389/fnhum.2015.00682)
Supplement: Supplementary file 1 [file DataSheet1.PDF]

Complementary Data

We ran a repeated measures ANOVA separately for each ISI comparing the left and right hemisphere lateral sites. We included the between subjects factor Language Group (monolingual, bilingual), and the within subjects factors Hemisphere (left, right), Context (dominant, subordinate), Target (dominant, subordinate, unrelated), site (F3/F4, FC3/FC4, C3/C4, CP3/CP4, P3/P4), and Time (300-600 ms post-stimulus divided into 50 ms intervals). There was a main effect of Hemisphere at both the short ($F(1,66)=11.2$, $MSE=541.1$, $p<.01$, $\varepsilon=1.0$) and long ($F(1,66)=13.0$, $MSE=229.3$, $p<.01$, $\varepsilon=1.0$) ISIs, showing that N400 amplitude was larger over the left hemisphere. There was one significant interaction involving the factors Hemisphere and Language Group, however simple effects analyses showed that this was not a systematic difference between monolinguals and bilinguals. Specifically, there was a significant Language Group x Hemisphere x Context x Time interaction ($F(5,330)=2.2$, $MSE=1.0$, $p=.02$, $\varepsilon=.71$), which showed no significant simple effects of language group or context, and N400 amplitude was larger over the left than the right hemisphere for both language groups. There were no other significant interactions involving Language Group and Hemisphere (all $p$s > .10). However, there was a Hemisphere x Context x Target x Site x Time interaction at the long ISI ($F(40,2640)=2.4$, $MSE=0.328$, $p=.01$, $\varepsilon=.26$) and a trend at the short ISI ($F(40,2640)=1.8$, $MSE=0.340$, $p=.06$, $\varepsilon=.30$). Simple effects analyses showed that the Context x Target pattern did not differ across the two Hemispheres.

In addition, we ran two (one for each hemisphere) Language Group (monolingual, bilingual) x Context (dominant, subordinate) x Target (dominant, subordinate, unrelated) x site (F3, FC3, C3, CP3, P3 / F4, FC4, C4, CP4, P4) x Time (300-600 ms post-stimulus

divided into 50 ms intervals) repeated measures ANOVAs to examine any effects of bilingualism in the laterals electrode sites. These analyses showed a similar pattern of results for both left and right lateral sites and replicated the findings from the analysis of midline sites. Of note is that the significant Language Group x Context x Target x Site interaction observed in the analysis of the midline sites was only significant in the analysis of right hemisphere lateral sites ($F(8,288)=2.9$, $MSE=6.4$, $p=.02$, $\varepsilon=.53$) and replicated the pattern seen in midline sites (i.e., bilinguals showed similar activation of both meanings of the homonym following a subordinate biasing context, whereas monolinguals showed less activation of the dominant than the subordinate meaning. Both groups showed a similar pattern following a dominant biasing context).
