# Supplementary material for: A Behavioral and Electrophysiological Investigation of the Effect of Bilingualism on Lexical Ambiguity Resolution in Young Adults
Source: Front Hum Neurosci. 2015 Dec 21;9:682. doi: 10.3389/fnhum.2015.00682 (PMC4685109; doi:10.3389/fnhum.2015.00682)
Supplement: Supplementary file 2 [file DataSheet2.docx]

Appendix 1

| HOMONYM | DOMINANT TARGET | SUBORDINATE TARGET |
| --- | --- | --- |
| ARMS | legs | guns |
| BALL | soccer | reception |
| BANK | money | river |
| BAR | drink | soap |
| BARK | dog | shell |
| BASE | home | bottom |
| BAT | ball | animal |
| BATTERY | power | assault |
| BEAM | ray | support |
| BLUFF | lie | cliff |
| BOARD | committee | plank |
| BOIL | bubble | infection |
| BREAK | rest | crack |
| BRUSH | hair | forest |
| BULB | lamp | flower |
| CABLE | television | rope |
| CALF | cow | leg |
| CAPITAL | city | asset |
| CHECK | mark | cash |
| CHEST | torso | treasure |
| CHIP | potato | nick |
| CLIP | fastener | trim |
| CLOG | block | shoe |
| COLD | warm | flu |
| COMPANY | business | companions |
| CORN | cob | foot |
| COURSE | class | path |
| CRICKET | insect | sport |
| CUE | pole | signal |
| DIAMOND | gem | baseball |
| DOWN | up | feather |
| DRILL | tool | practice |
| DROP | slope | molecule |
| DUCK | quack | bend |
| EXPRESS | fast | show |
| FALL | tumble | autumn |
| FAN | air | devotee |
| FAST | slow | starve |
| FENCE | barrier | sword |
| FILE | folder | buffer |
| FILM | cinema | picture |
| FIRE | match | gun |
| FIRM | solid | company |
| FLAT | level | apartment |
| FLIGHT | airplane | stairs |
| FLUSH | toilet | card |
| FOOT | toe | inch |
| GAME | play | moose |
| GEAR | sprocket | equipment |
| GRAVE | tomb | serious |
| GROUND | floor | mash |
| HABIT | addiction | attire |
| HIDE | seek | pelt |
| HORN | honk | antler |
| JAM | jelly | backup |
| JET | plane | geyser |
| JOINT | knee | connection |
| KIND | nice | type |
| LACE | frill | string |
| LAP | seat | swim |
| LEAN | skinny | tilt |
| LEFT | right | remaining |
| LETTER | envelope | alphabet |
| LIGHT | lantern | heavy |
| LIP | mouth | rim |
| LITTER | trash | kitten |
| LOCK | key | canal |
| LOG | wood | journal |
| MAJOR | army | minor |
| MARBLE | tile | glass |
| MATCH | fire | event |
| MEAN | nasty | average |
| MINE | yours | gold |
| MINT | leaf | coin |
| MODEL | fashion | design |
| MOLD | fungus | shape |
| NAIL | hammer | finger |
| NOTE | message | music |
| NOVEL | story | original |
| ORDER | request | organization |
| PAGE | book | boy |
| PALM | fist | tree |
| PANEL | board | judge |
| PARTY | dance | coalition |
| PATIENT | doctor | tolerant |
| PEN | pencil | cage |
| PERCH | branch | fish |
| PIPE | smoke | water |
| PIT | hole | seed |
| PITCH | hit | tone |
| PLAIN | ordinary | field |
| PLANE | fly | surface |
| PLANT | pot | factory |
| PLAY | fun | theatre |
| POKER | cards | stick |
| PORT | boat | wine |
| POST | mail | station |
| PRESENT | gift | future |
| PRUNE | plum | cut |
| PUPIL | student | eye |
| RACE | competition | nationality |
| RACKET | tennis | noise |
| RASH | red | reckless |
| REEL | rod | movie |
| RING | wedding | bell |
| ROLL | over | bun |
| ROW | oar | column |
| RULER | measure | king |
| SCALE | balance | skin |
| SCREEN | monitor | mosquito |
| SEASON | spring | salt |
| SECOND | first | minute |
| SENTENCE | phrase | ruling |
| SHIFT | gear | work |
| SHOOT | hurt | sprout |
| SINK | tub | float |
| SLIDE | park | specimen |
| SLIP | glide | dress |
| SNAP | tear | button |
| SPADE | shovel | diamond |
| SQUASH | raquetball | vegetable |
| STAFF | employee | cane |
| STALK | hunt | stem |
| STALL | stable | stop |
| STAND | sit | podium |
| STAR | sky | actor |
| STATE | province | condition |
| STATIC | cling | motionless |
| STERN | strict | bow |
| STORE | shop | supply |
| STRAIN | stress | filter |
| STROKE | golf | heart |
| STUD | hunk | wall |
| SUIT | tie | trial |
| SWALLOW | gulp | bird |
| TENDER | loving | currency |
| TERM | session | word |
| TIE | neck | draw |
| TIRE | wheel | bore |
| TOAST | bread | tribute |
| TOLL | fee | chime |
| TOP | summit | spin |
| TRAIN | railroad | gown |
| TRIAL | court | test |
| TRIP | vacation | stumble |
| TRUNK | case | snout |
| TYPE | write | kind |
| VESSEL | ship | vein |
| WATCH | clock | see |
| WAVE | ocean | hand |
